# Cultural activity participation and associations with self-perceived health, life-satisfaction and mental health: the Young HUNT Study, Norway

**DOI:** 10.1186/s12889-015-1873-4

**Published:** 2015-06-10

**Authors:** Elisabeth Hansen, Erik Sund, Margunn Skjei Knudtsen, Steinar Krokstad, Turid Lingaas Holmen

**Affiliations:** HUNT Research Centre, Department of Public Health and General Practice, Faculty of Medicine, Norwegian University of Science and Technology, Forskningsveien 2, 7600 Levanger, Norway; Department of Health Promotion, Nord-Trøndelag County Council, Seilmakersgata 2, Steinkjer, 7735 Norway; Psychiatric Department, Levanger Hospital, North-Trøndelag Health Trust, Kirkegata 2, Levanger, 7600 Norway; Department of Health Sciences, Mid Sweden University, Kunnskapens väg 8, 83125 Östersund, Sweden

**Keywords:** Culture activity, Adolescents, Mental health, Self-perceived health, Self-esteem, Life-satisfaction, Anxiety and depression

## Abstract

**Background:**

Leisure time activities and culture participation may have health effects and be important in pulic health promotion. More knowledge on how cultural activity participation may influence self-perceived health, life-satisfaction, self-esteem and mental health is needed.

**Methods:**

This article use data from the general population-based Norwegian HUNT Study, using the cross-sectional Young-HUNT3 (2006–08) Survey including 8200 adolescents. Data on cultural activity participation, self-perceived health, life-satisfaction, self-esteem, anxiety and depression were collected by self-reported questionnaires.

**Results:**

Both attending meetings or training in an organisation or club, and attending sports events were positively associated with each of the health parameters good self-percieved health, good life-satisfaction, good self-esteem, and low anxiety and depression symptoms. We found differences according to gender and age (13–15 years versus 16–19 years old) for several culture activities, where girls aged 16–19 years seemed to benefit most from being culturally active. The extent of participation seemed to matter. Those who had frequent participation in cultural activities reported better health outcomes compared to inactive adolecents.

**Conclusions:**

The results from this study indicate that participation in cultural activities may be positively associated with health, life-satisfaction and self-esteem in adolescents and thus important in public health promotion. Possible sex and age differences should be taken into account.

**Electronic supplementary material:**

The online version of this article (doi:10.1186/s12889-015-1873-4) contains supplementary material, which is available to authorized users.

## Background

Increasing prevalence of disability in young adults and persisting inequalities in health illustrates a need for innovative preventive strategies. A recent scientific paper from the Norwegian HUNT Study, reported that cultural attendance was associated with better health, quality of life and lower levels of anxiety and depression in adults suggesting that this may be utilized in health promotion [1]. In some countries governments see a potential for a policy of using culture activities to stimulate participation in society based on the assumption that this will improve health [2]. The culture concept may be understood as arts and literature, but may also include lifestyle, ethics and tradition [3]. This suggests that cultural activities may be of importance in public health strategies also concerning mental health, and urge the need for further studies in this public health topic also into younger populations. Cultural capital are by Bourdieu [4] defined as people’s symbolic and informational resource for action. Health-related cultural capital might be regarded as cultural resources which are important and should be available to people to maintain and prolong good health, knowledge and practical skills [5].

Health effects from physical activity are well documented both in youth and adulthood [6, 7]. Psycho-physiological approaches including music, experiences of music and dancing also seem to have positive health effect [8, 9], and Hansen et al. [10] found that social relations are of significance to motivational health-related behavior. This is in line with studies where social participation (cultural, religious, social activities) was found to be associated with survival, longevity and where also participation in cultural activities might have a positive influence and personal development [11–13]. Still there is a lack of research on possible health effects of other, or more specific, cultural activities. Research on cultural activity and associations with health have to be investigated in terms of a defined knowledge or understanding of what kind of specific cultural activity do we want to achieve knowledge about (e.g., reading book, playing an instrument, audience at sports events) [14]. Additionally, if cultural activities are to be used for achieving good health or other social goals, it is of importance to know for whom, what kinds of activities that might have effect, what extent that is necessary, and what motivates participation in such activity [15, 16]. There is a need to establish a firm empirical basis for intervention programs to promote health and well-being. The field of epidemiology traditionally focuses on risk factors of illness. However Theorell and Kreuts [9] suggest that epidemiological approaches in addition should identify factors that might protect against ill health by enhancing immune functions and actively promote health.

Children with disabilities may experience different obstacles in school and support systems, which again might influence patterns of participation in leisure time activities [17]. It is likely that positive habits acquired during childhood and youth might prevent adverse behavior later in life and have a positive impact on health. However there is still lack of knowledge regarding the health benefits of cultural attendance [15] and what mechanisms might underpin these relationships, especially in adolescence and from general population based studies [2].

Our first aim was to explore if self-reported leisure time cultural activity participation, (e.g., playing an instrument, being at a meeting or training in an organisation or club, making film, being audience at sports events, cinema), was associated with subjective health in an adolescent population. More specifically, we study associations with self-perceived health, life-satisfaction, self-esteem, anxiety and depression among adolescents. Secondly the aim was to investigate any dose response regarding the associations between extents of self-reported leisure time cultural activity and the same health parameters. The Norwegian Young HUNT Study [18] is a large scale population based study of an entire adolescent population, and are utilized to answer these questions.

## Methods

### Study population

The Nord-Trøndelag Health Study (HUNT) is a total population based study in the County of Nord-Trøndelag, Norway [19]. Nord-Trøndelag County has a geographical, demographical and occupational structure fairly representative of Norway as whole [19, 20].

### The young-HUNT data collection

The Young-HUNT Study is the adolecent part (13–19 years) of the HUNT Study including all students in junior and senior High Schools (lower and upper Secondary Schools) in the county [18] In the present study data from 8200 adolescents (78,4 % of the invited) attending the Young-HUNT3 Survey (2006–08) were used. The students completed a comprehensive questionnaire during one school hour in an exam situation. The Young-HUNT3 questionnaire included data on lifestyles, somatic and mental health problems, well-being and leisure time activities.

### Measures

#### Culture participation

A total of 8085 participants (4074 girls and 4011 boys) completed the questions on cultural activity participation. Culture activity was assessed by the head question: How often have you done any of these activities in the past week?: Read a book/magazine/comic book, listened to music, played an instrument, was at a meeting or training with a club/team, was at the library, went to the movies, was in a play/theatre, did photography/film, went to a concert, went to watch a sport event/game, sang in a chore. Values from 1 to 4 for each answer category regarding frequency were assigned “None”, “Once”, “2-3 times”, “4 times or more” in the last 7 days.

The activities were dichotomised into Low (0) and High (1) cultural activity by normative cut off for each question included. Read a book/magazine/comic book; played an instrument: Low = None, and Once, High = 2-3 times, 4 times or more. Listened to music: Low = None, Once, High = 2-3 times, 4 times or more. Was at a meeting or training with a club/team; was at the library; went to the movies; was in a play/theatre; did photography/film; went to a concert; went to watch a sport event/game; sang in a chore. Low = None, High = Once, 2-3 times, 4 times or more.

Further the culture activity questions were summed (totally 11) into a total culture scale index and divided into three categories of extent (low active = 0–2, medium active = 3–4, and high active 5–11).

### Covariates

*Socio economy* was assesed by the question: How well off do you think your family is compared to most others? Response alternatives were: “about the same as most others, better financial situation, worse financial situation”. In the stratisfied analyses we dichotomized the socio-economic variable into High (1) positive family economy (about the same as most others, better financial situation), and Low (0) family economy (worse financial situation).

### Outcome variables

*Perceived health (PH)* [21] was assessed by the question: “How is your health at the moment?”. Response alternatives were: “poor, not so good, good, very good”. The perceived health variable was dichotomized into one category for poor self-perceived health (not so good and poor), and good self-perceived health (very good and good).

*Life-Satisfaction*. The subjects were in the questionnaire asked: “Thinking about your life at the moment, would you say that you by and large are satisfied, or are you mostly dissatisfied?”. Response alternatives were: very satisfied, satisfied, somewhat satisfied, neither satisfied nor dissatisfied, somewhat dissatisfied, dissatisfied, very dissatisfied. The variable was dichotomized into one category for Low life-satisfaction (neither satisfied nor dissatisfied, somewhat dissatisfied, dissatisfied, very dissatisfied), and second category High life-satisfaction (very satisfied, satisfied, somewhat satisfied).

*Self-esteem* was assessed by four out of ten questions of Rosenberg’s scale [22, 23]. The questions were “How do you see yorself?: I take a positive attitude toward myself, I certainly feel useless at times, I feel I do not have much to be proud of, I feel that I’m a person of worth, at least on an equal plane with others. Response alternative were: strongly agree, agree, disagree, strongly disagree. The index scale variable by self-esteem was computed with the four questions together. Two of the questions response alternatives were reversed in order to have all answering alternatives going in the same direction. Cronbach’s alpha for self-esteem was .771, Scala range: 4–16, and for statistical analyses as logistic regression later dichotomized into category Low self-esteem (4–8), and category of High self-esteem (9–16).

*Anxiety and depression.* For measuring psychological factors to assess anxiety and depression symptoms, a 5-item version of Symptom Check-list (SCL-5) proven reliable in previous studies [24, 25] was used. The index for this category was assessed by the head question: In the last 14 days, have you been bothered by any of these?: You have been constantly afraid and anxious, You have felt tense or uneasy, You have felt hopelessness when you think of the future, You have felt dejected or sad, You have worried too much about various things. Response alternatives were: 1 = Not bothered, 2 = a little bothered, 3 = quite bothered, 4 = very bothered. Max score 20, range 5 to 20. Cronbach’s alpha for SCL-5 was .829. For statistical analyses as logistic regression the response alternatives were dichotomized into category for High anxiety/depression (5–14), and category for Low anxiety/depression (15–20).

### Statistical analysis

Background descriptive analyses of data are reported as percentages (%) and were performed prior to the specification of logistic regression analyses. The regression analysis was stratified according to age and gender and associations was examined for each cultural activity according to the four outcomes (self-perceived health, life-satisfaction, self-esteem and anxiety/depression) in unadjusted models (model 1). We also specified models adjusting for socioeconomic status (model 2). Similarily stratified regression models were specified between the cultural activity index and the four outcomes. All effects are reported as odds ratio (OR) with 95 per cent confidence intervals (95 % CI). IBM SPSS version 21 was used for the statistical analyses and Microsoft Excel 2010 and Forest Plot viewer were used to produce tables and plots.

### Ethics

The respondents in the Young-HUNT 3 Survey participated voluntary. The survey was approved by Norwegian Data Inspectorate, The Directorate of Health, and by the Regional Committee for Medical Health Research Ethics (REC). All participants and in addition the parents of adolescents younger than 16 year of age signed an informed consent. This specific study was also approved by REC.

## Results

Descriptive background statistics (Tables 1 and 2) presents an overview of number of participants, girls and boys 13–15 years and 16–19 years old. Variations in number of respondents, percentage participation of each culture activity, each health parameter and family economy.

**Table 1:** Participants reporting positive family economy, good self-perceived health, good life-satisfaction, good self-esteem and, low anxiety and depression (lower secondary school (13–15 years), upper secondary school (16–19 years)). The Young-HUNT Study 2006–08

	Girls		Boys	
	13-15years	16-19years	13-15years	16-19years
	*N* = 2357	*N* = 1717	*N* = 2392	*N* = 1619
Pos. family economy	90.5 %	89.5 %	93.0 %	89.6 %
Good self-perceived h.	90.7 %	83.9 %	91.6 %	89.2 %
Good life-satisfaction	67.6 %	67.9 %	83.4 %	83.6 %
Good self-esteem	87.9 %	88.4 %	96.6 %	96.7 %
Low anxiety & depr.	84.2 %	75.6 %	94.9 %	91.4 %

Variations in number of respondents (n) and percentage participation (%). Missing < 5 %

**Table 2:** Reported culture activity the last week among boys and girls 13–15 years and 16–19 years

	Girls		Boys	
	13-15years	16-19years	13-15years	16-19years
	*N* = 2357	*N* = 1717	*N* = 2392	*N* = 1619
Reading book	49.1 %	43.7 %	47.5 %	39.1 %
Listen to music	77.8 %	84.2 %	68.2 %	82.4 %
Playing instrument	21.5 %	13.6 %	25.3 %	21.6 %
Meeting/training	67.1 %	51.5 %	61.4 %	53.9 %
Visiting library	24.7 %	30.4 %	22.6 %	27.3 %
Visiting cinema	18.0 %	14.7 %	20.8 %	21.8 %
Playing theatre	6.9 %	4.9 %	5.8 %	4.6 %
Film/photo	22.6 %	19.9 %	16.9 %	21.0 %
Music event	12.9 %	8.1 %	13.2 %	10.8 %
Sports event	28.5 %	23.8 %	23.5 %	26.4 %
Singing choir	8.1 %	6.0 %	3.8 %	5.5 %

Variations in number of respondents (n), percentage participation (%) in high culture activity. Missing < 5 %

Overall, we did not find any clear pattern in which all kinds of cultural activities was associated with good health in this material. However, some types of activities systematically showed positive associations. Both participating at a meeting or training in an organisation or club, and being audience at sports events were positively associated with each of the health parameters. We found differences according to gender and age, where girls seemed to benefit most from being culturally active. Those who had participation in cultural activities reported better health outcomes compared to low or inactive adolecents.

### Cultural activites and health in adolescence

#### Perceived health

Being culture active by meeting or training in an organisation or club, seems to positively be associated with good perceived health. In girls in lower secondary school the OR was = 2.90 (CI = 2.16 to 3.90), and in upper secondary school OR = 2.47 (CI = 1.87 to 3.28). For boys the corresponding ORs were OR = 2.75 (CI = 2.01 to 3.76) and OR = 3.21 (CI = 2.25 to 4.58) (Additional file 1: Figure S1). The same pattern was found regarding reporting being audience at sports events in girls in lower secondary school OR = 2.17 (CI = 1.48 to 3.20) and in upper secondary school OR = 2.61 (CI = 1.76 to 3.87), and for boys in upper secondary school OR = 3.37 (CI = 2.01 to 5.65).

#### Life- satisfaction, Self-esteem, Anxiety and depression

For these health measures tha data showed similar patterns (Additional file 1: Figure S2, S3, S4). Boys had however less positive associations towards self-esteem and, anxiety and depression compared to girls. Additionally, girls in upper secondary school had a positive association between visiting cinema and good life-satisfaction OR = 1.62 (CI = 1.14 to 2.30), and girls in lower secondary school had a positive association between playing an instrument and good self-esteem OR = 1.62 (CI = 1.14 to 2.30).

### Cultural activites and health in adolescence, adjusted for socio-economic status

The significant associations between culture activites and health parameters described above (illustrated in Additional file 1: Figure S1–S4), were adjusted for socio-economic status (family economy) (Table 3). *Self-perceived health* showed the strongest association with cultural activities. To some extent there were also positive associations towards the other health aspects (life-satisfaction and self-esteem), while anxiety and depression showed the weakest association.

**Table 3:** Association between selected cultural activities and good self-perceived health, good life-satisfaction, good self-esteem, and low anxiety & depression, adjusted for socio-economic status in model II. Only significant associations are included. OR ref.value: Not so good/high anxiety & depression = 1.0

	OR (95 % CI)	OR (95 % CI)	OR (95 % CI)	OR (95 % CI)
	Model I		Model II	
	Girls	Boys	Girls	Boys
Good Self-perceived health:				
Ref. value:	1.0	1.0	1.0	1.0
	Meeting/training		Meeting_training/Fam.eco	
13-15 years	2.90 (2.16 3.90)	2.75 (2.01 3.76)	2.70 (2.00 3.66)	2.80 (2.02 3.88)
16-19 years	2.47 (1.87 3.28)	3.21 (2.25 4.58)	2.43 (1.83 3.24)	3.07 (2.14 4.41)
	Sports event		Sports event/Fam.eco	
13-15 years	2.17 (1.48 3.20)		2.12 (1.43 3.12)	
16-19 years	2.61 (1.76 3.87)	3.37 (2.01 5.65)	2.59 (1.74 3.87)	3.24 (1.90 5.53)
Good SatLife:				
Ref. value:	1.0	1.0	1.0	1.0
	Meeting/training		Meeting_training/Fam.eco	
13-15 years	1.67 (1.39 2.01)	1.74 (1.38 2.19)	1.57 (1.30 1.91)	1.71 (1.35 2.17)
16-19 years	2.61 (1.76 3.87)	3.37 (2.01 5.65)	1.66 (1.34 2.06)	1.71 (1.29 2.27)
	Visiting Cinema		Visiting cinema/Fam.eco.	
16-19 years	1.48 (1.08 2.02)		1.53 (1.11 2.12)	
	Sports event		Sports event/Fam.eco.	
13-15 years	1.52 (1.23 1.86)	1.42 (1.07 1.90)	1.52 (1.23 1.88)	1.43 (1.06 1.93)
16-19 years	1.74 (1.34 2.26)		1.69 (1.29 2.20)	
Good Self-esteem:				
Ref. value:	1.0	1.0	1.0	1.0
	Playing instrument		Playing instrument/Fam.eco.	
13-15 years	1.62 (1.14 2.30)		1.60 (1.12 2.28)	
	Meeting_training		Meeting_training/Fam.eco.	
13-15 years	1.91 (1.47 2.48)	1.79 (1.11 2.89)	1.76 (1.34 2.30)	1.61 (0.98 2.63)
16-19 years	1.73 (1.27 2.36)		1.67 (1.22 2.28)	
	Sports event		Sports event/Fam.eco.	
13-15 years	1.44 (1.06 1.95)		1.43 (1.04 1.95)	
16-19 years	2.02 (1.32 3.09)		1.92 (1.25 2.95)	
Low anxiety/depression:				
Ref. value:	1.0	1.0	1.0	1.0
	Meeting_training		Meeting_training/Fam.eco.	
13-15 years	1.41 (1.11 1.79)	1.48 (1.01 2.18)	1.27 (0.99 1.62)	1.36 (0.91 2.03)
16-19 years	1.40 (1.12 1.76)		1.40 (1.11 1.77)	
	Sports event		Sports event/Fam. eco.	
16-19 years	1.88 (1.40 2.53)		1.86 (1.38 2.52)	

13-15 years old = lower secondary school; 16–19 years old = upper secondary school

**Table 4:** Cultural activity index indicating medium and high participation compared to low (inactive) rate in 13–15 years olds and 16–19 years olds (girls, boys) associated with good self-perceived health, good life-satisfaction, good self-esteem, low anxiety & depression, adjusted for socio-economic status in model II. Reference value OR = 1.0 for low active (inactive)

	OR (95 % CI)	OR (95 % CI)	OR (95 % CI)	OR (95 % CI)
	**Model 1:**		**Model 2:**	
	**Medium and high active**		**Model 1 adj. for Fam. economy vs. low active**	
	**Girls**	**Boys**	**Girls**	**Boys**
**Good Self-perceived health:**				
**Ref. value:**	**1.0**	**1.0**	**1.0**	**1.0**
13-15 years Med.	**1.41 (1.01 1.98)**	**1.70 (1.18 2.45)**	**1.42 (1.01 1.20)**	**1.67 (1.16 2.42)**
13-15 years High	1.40 (0.93 2.09)	1.16 (0.74 1.80)	1.36 (0.90 2.04)	1.16 (0.74 1.81)
**Ref. value:**	**1.0**	**1.0**	**1.0**	**1.0**
16-19 years Med.	**1.90 (1.41 2.57)**	1.31 (0.91 1.88)	**1.85 (1.36 2.50)**	1.31 (0.91 1.90)
16-19 years High	**1.91 (1.25 2.90)**	**1.92 (1.15 3.20)**	**1.85 (1.21 2.82)**	**2.05 (1.22 3.46)**
**Good SatLife:**				
**Ref. value:**	**1.0**	**1.0**	**1.0**	**1.0**
13-15 years Med	1.07 (0.87 1.32)	1.08 (0.84 1.40)	1.09 (0.88 1.35)	1.06 (0.81 1.38)
13-15 years High	1.25 (0.97 1.60)	0.97 (0.70 1.35)	1.20 (0.93 1.56)	1.04 (0.74 1.48)
**Ref. value:**	**1.0**	**1.0**	**1.0**	**1.0**
16-19 years Med	**1.42 (1.13 1.78)**	1.02 (0.75 1.38)	**1.37 (1.08 1.73)**	1.03 (0.75 1.41)
16-19 years High	**1.43 (1.05 1.96)**	0.86 (0.60 1.25)	**1.47 (1.07 2.02)**	0.93 (0.64 1.37)
**Good Self-esteem:**				
**Ref. value:**	**1.0**	**1.0**	**1.0**	**1.0**
13-15 years Med	1.03 (0.77 1.38)	1.47 (0.87 2.47)	1.05 (0.77 1.41)	1.34 (0.79 2.29)
13-15 years High	1.32 (0.91 1.91)	2.19 (0.97 4.97)	1.27 (0.87 1.84)	2.08 (0.91 4.76)
**Ref. value:**	**1.0**	**1.0**	**1.0**	**1.0**
16-19 years Med	**2.08 (1.47 2.95)**	0.99 (0.53 1.86)	**1.98 (1.40 2.83)**	1.04 (0.54 1.99)
16-19 years High	**1.75 (1.10 2.78)**	1.05 (0.47 2.35)	**1.66 (1.04 2.65)**	1.28 (0.55 2.99)
**Low anxiety/depression:**				
**Ref. value:**	**1.0**	**1.0**	**1.0**	**1.0**
13-15 years Med	0.95 (0.72 1.24)	0.62 (0.40 0.97)	0.94 (0.71 1.24)	0.58 (0.37 0.91)
13-15 years High	0.89 (0.65 1.21)	0.69 (0.39 1.22)	0.85 (0.62 1.18)	0.72 (0.40 1.31)
**Ref. value:**	**1.0**	**1.0**	**1.0**	**1.0**
16-19 years Med	1.15 (0.90 1.48)	0.75 (0.50 1.14)	1.12 (0.86 1.44)	0.74 (0.48 1.14)
16-19 years High	1.03 (0.74 1.43)	0.75 (0.45 1.24)	1.01 (0.72 1.41)	0.86 (0.50 1.47)

13-15 years Med. = lower secondary school, medium culture active; 13–15 years High = lower secondary school, high culture active. 16–19 years Med. = upper secondary school, medium culture active; 16–19 years High = upper secondary school, high culture active. Bold values indicate significant associations

### Extent of cultural activites and health

The data suggested no consistent dose response association between extent for cultural activity and health outcomes. No significant positive associations were found for adolescents in lower secondary school between the culture activity index and life-satisfaction, self-esteem, or low anxiety and depression, except for self-perceived health.

#### Perceived health

Results indicated, after adjusted for family economy, that girls in lower secondary school being medium cultural active had an OR = 1.42 (CI = 1.01 to 1.20) and boys OR = 1.67 (CI = 1.16 to 2.42) for good self-perceived health. Also girls in upper secondary school being medium culture active had an OR = 1.85 (CI = 1.36 to 2.50), the highly culture active an OR = 1.85 (CI = 1.21 to 2.82) and boys in upper secondary school being highly culture active an OR = 2.05 (CI = 1.22 to 3.46) for good self-perceived health.

#### Life- satisfaction

Cultural activity were associated with good life-satisfaction for most adolescence 16–19 years. Girls beung medium active OR = 1.37 (CI = 1.08 to 1.73), and highly active OR = 1.47 (CI = 1.07 to 2.02).

#### Self-esteem

Regarding good self-esteem, girls in upper secondary school being cultural medium active had an OR = 1.98 (CI = 1.40 to 2.83), and the highly active an OR = 1.66 (CI = 1.04 to 2.65).

#### Anxiety and depression

Analyses between the culture activity index and anxiety and depression revealed no significant associations (Table 4).

## Discussion

This is, to our knowledge, the only large-scale population based study assessing adolescent’s cultural activity in relation to self-perceived health, life-satisfaction health, self-esteem health, and anxiety and depression. We did not find any clear pattern in which all kinds of cultural activities was associated with good health in this material or a consistent dose response pattern. However, the data revealed that some cultural aspects were associated positively with health. These results are partly in line with findings from a Swedish investigation including culture and health [26], were the relation between different kinds of cultural activities could be associated with subjective health. The results from the present study highlight two cultural activities in particular. Both participating at a meeting or training in an organisation or club, and being audience at sports events were positively associated with all of the investigated health outcomes (self-perceived health, life-satisfaction, self-esteem, and anxiety and depression). This suggest that attendance at cultural events like sport and social happenings might be important for of adolescent health and social goals in the future, and are in line with Turpin’s [15] interpretation of how cultural activities and art may be used for achieving other social goals. Still there is yet to prove if this associations is caused by physical activity rather than the subjective positive experiences by cultural participation [26]. It is interesting that cultural participation seems more important for adolescent girls by showing a stronger association on health compared to boys at the same age. For girls and boys in lower secondary school (13–15 years) being medium cultural active was associated positively with self-perceived health. However, high cultural activity did not show this same positive association among the young adolescents. Might the level of time and engagement in cultural activities be too much and effect the youngest adolescent group negatively, while among the oldest age group the results indicate the opposite. One could reflect over signals indicating that in certain age spans during life the amount of participation may vary, and if the practice are not well balanced it might serve as a kind of stressor working against good health. This would though need further investigation. The data from the Young-HUNT used in this study showed a far less consistent association between reported cultural participation and health than the data for adults in the same population [1]. This may be related to the reported patterns of cultural activities for adults represent more stable habits than in adolescents. Adolescents probably change behavior more often than adults. It may also be that the health-related behavior that shows a clear positive association with health in this article, are behaviors that are more stable over time, or have a greater impact in the daily lives of the participants. An interesting result from the present study however, is the fact that cultural activity index did not show any associations with anxiety and depression in any of the age or gender groups. In terms of the biological mechanisms between cultural participation and health, several theories are described, and Johansson et al. [27] suggested that psycho-neuroimmunological theories may be relevant for understanding and explaining these effects. For instance, music may influence central physiological variables like blood pressure, heart rate, respiration, as well as immune and endocrine functions, which in turn promote health and longevity [28, 29]. Social relations [15, 16] might be one other possible explanation for positive pattern of effects from cultural activity participation. Literature in this area of research of cultural activity participation and associations to subjective health among adolescents are limited. Longitudinal designs and tracking studies of short-, medium- and long term health effects are in demand to assess the sustainability of culture effects across the life-span [9].

### Strengths, and Limitations of the study

This is a general population based study with a relatively large sample. Participants were blinded for the research hypothesis as the questions were asked in a general comprehensive health survey. The estimates were adjusted for confounding by socioeconomic status. The culture activity variables were collected from questions pointing back for a time span of one week. This is a more proper time frame for some aspects than others. This might however weaken the culture data as source for predicting health outcomes in adolescents. Some of the activities included in the culture activity variable scales might not have been available in the period before the questions were asked, giving misrepresentatives in the results. On the other hand, with the large number of cases included in the study in combination with statistical analyses compensating for such chrewiness, one could argue for a fairly reliable and valid information concerning the issue being discussed. In addition, we performed a large number of hypothesis tests, and the probability of seeing a significant difference increases with the number of tests performed. As a consequence, some of the proclaimed significant differences migth be Type 1 errors where we as a result of hypothesis testing rejected the null hypothesis of no difference, when in fact no difference exist in the population [30]. The cross-sectional design obviously preclude any causal claims, and the direction could go either way, good health might enhance cultural participation and vice versa.

## Conclusions

The present study especially endorse participating at a meeting or training in an organisation or club, and being audience at sports events for being positive associated with good health. This was found concerning anxiety and depression for both boys and girls in the younger age group, but for girls only at the age of 16–19 years. Having high compared to low cultural activity participation affected health related outcomes like self-perceived health, life-satisfaction and self-esteem positively, but had no effect on anxiety and depression. The data suggested gender and age differences as girls and the oldest age group seemed to benefit the most. Some cultural activities may be important to enhance health in adolecence.

## Additional file

Additional file 1: Figure S1. Good Self-perceived health in girls and boys (13–15 years and 16–19 years old). **Figure S2.** Good Life-satisfaction health in girls and boys (13–15 years and 16–19 years old). **Figure S3.** Good Self-esteem health in girls and boys (13–15 years and 16–19 years old). **Figure S4.** Low anxiety/depression in girls and boys (13–15 years and 16–19 years old).
