# Supplementary material for: Cultural activity participation and associations with self-perceived health, life-satisfaction and mental health: the Young HUNT Study, Norway
Source: BMC Public Health. 2015 Jun 10;15:544. doi: 10.1186/s12889-015-1873-4 (PMC4460785; doi:10.1186/s12889-015-1873-4)

**ADDITIONAL FILES**

**Figure 1. Good Self-perceived health in girls and boys (13-15 years and 16-19 years old).**


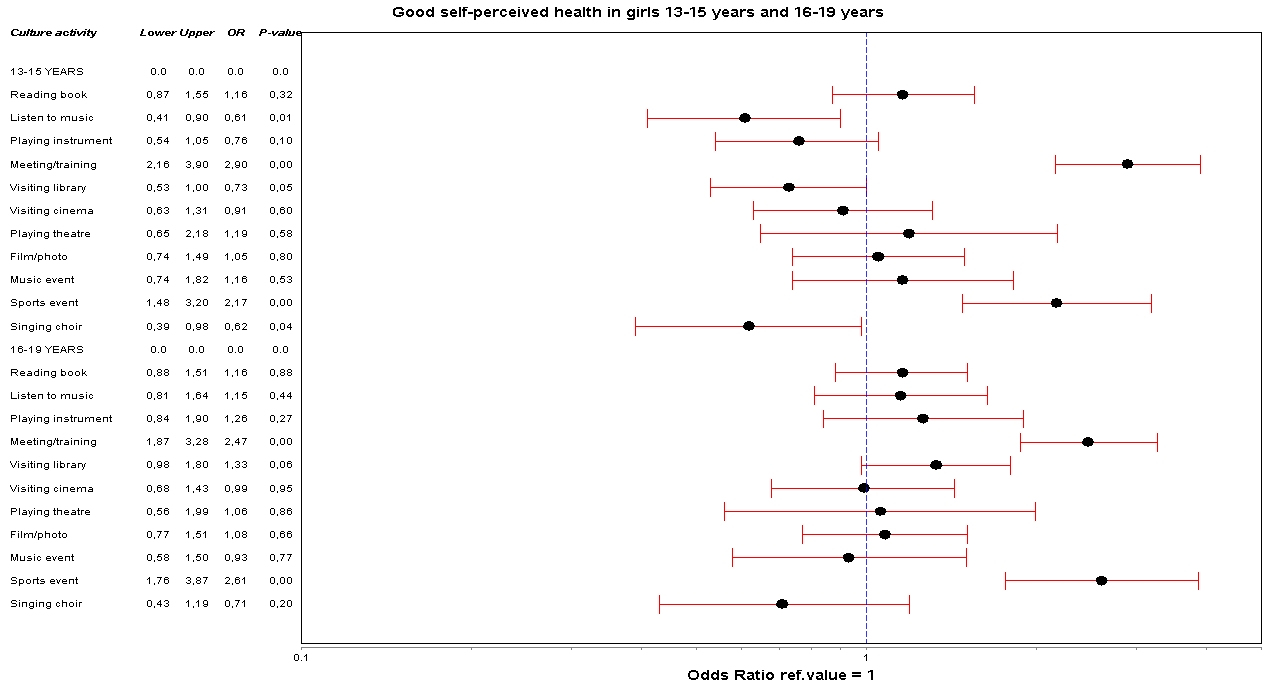


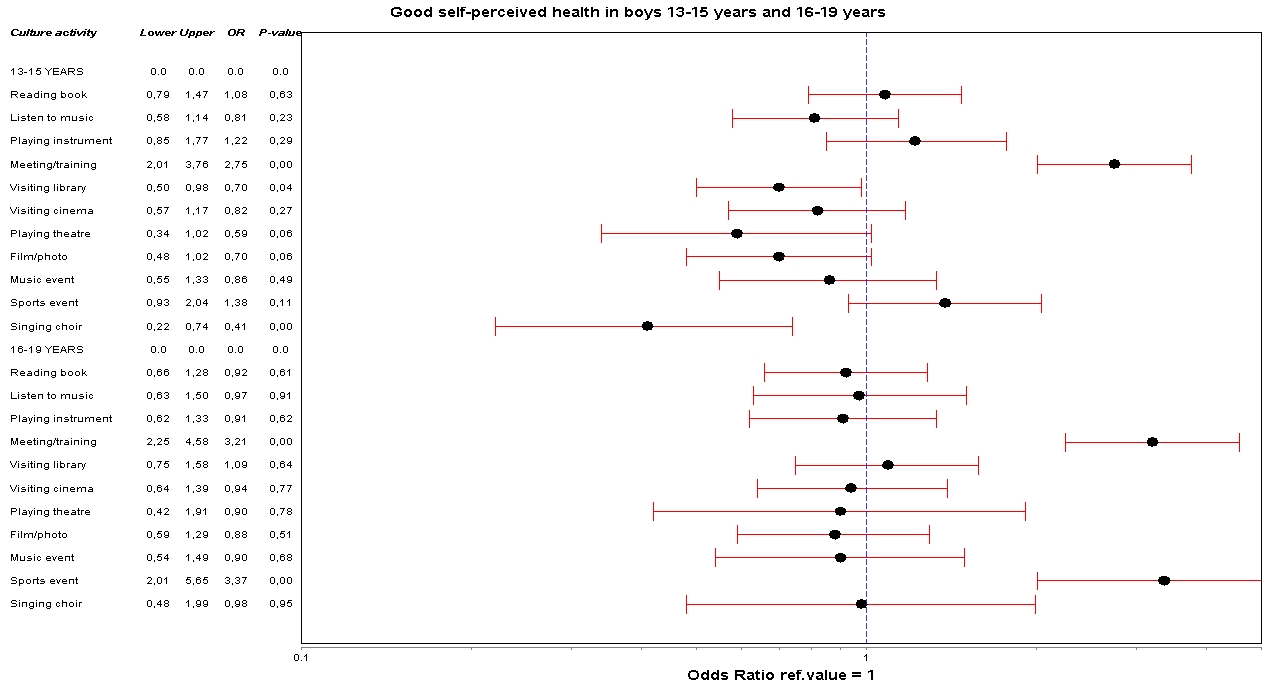


**Figure 2. Good Life-satisfaction health in girls and boys (13-15 years and 16-19 years old).**


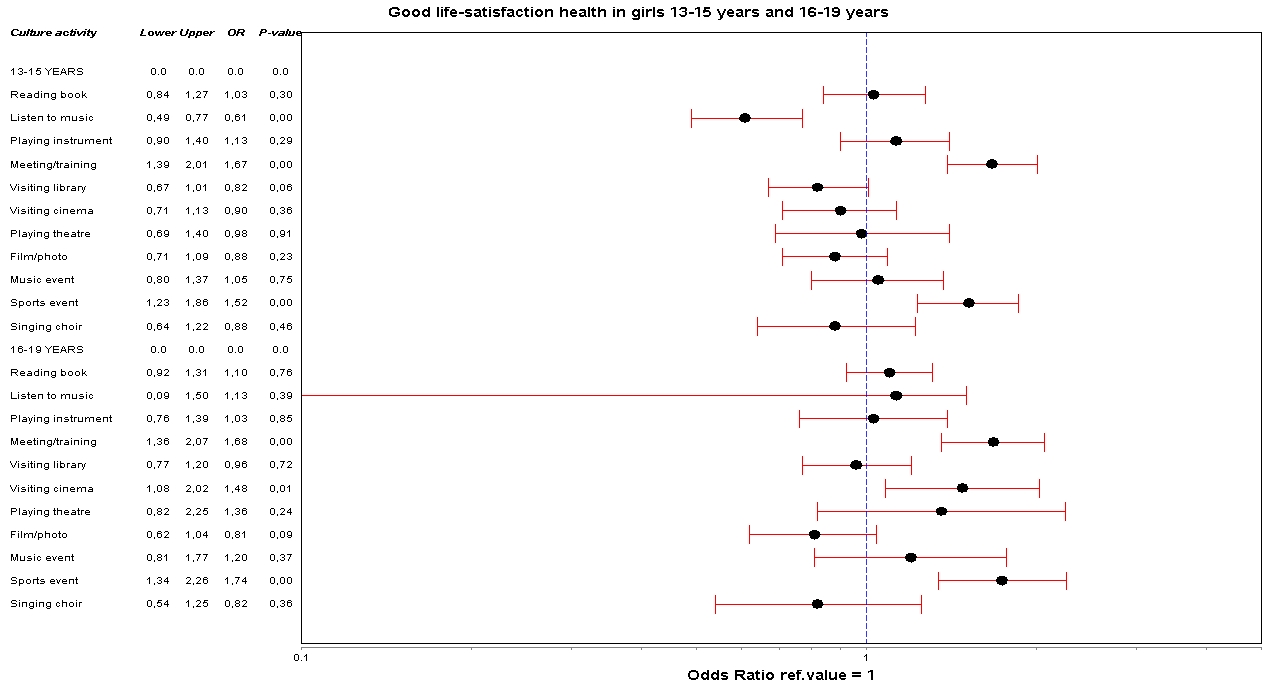


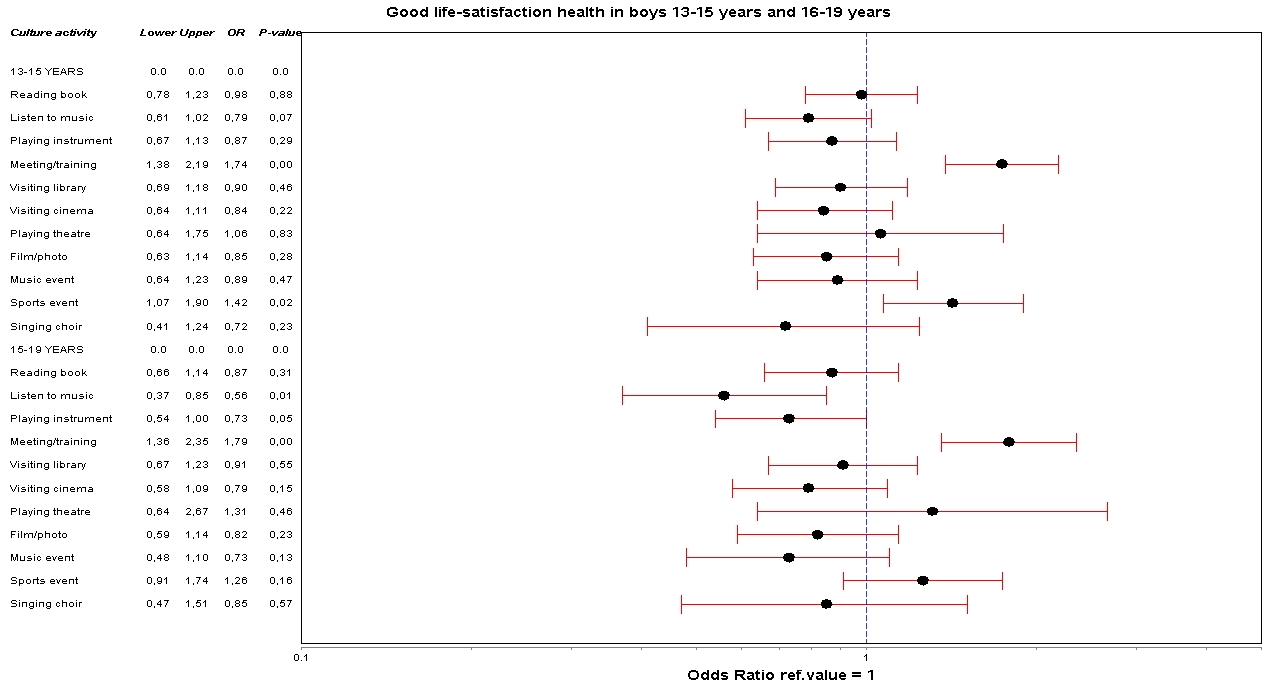


**Figure 3. Good Self-esteem health in girls and boys (13-15 years and 16-19 years old).**


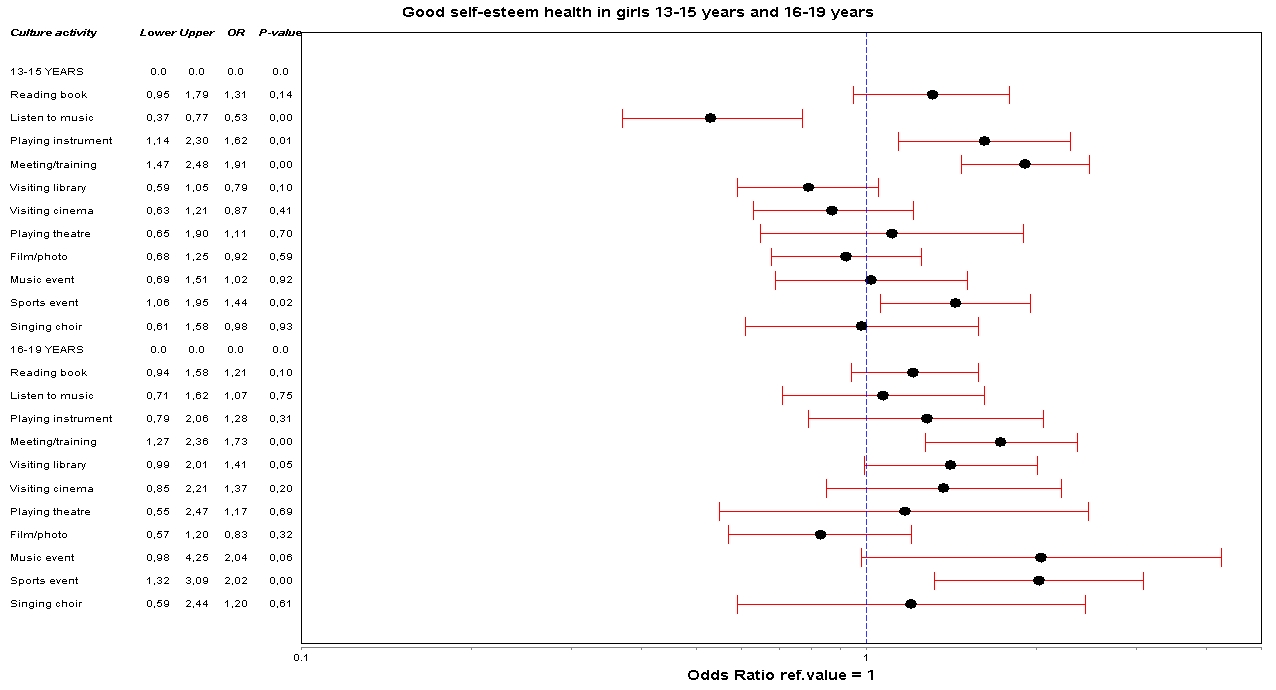


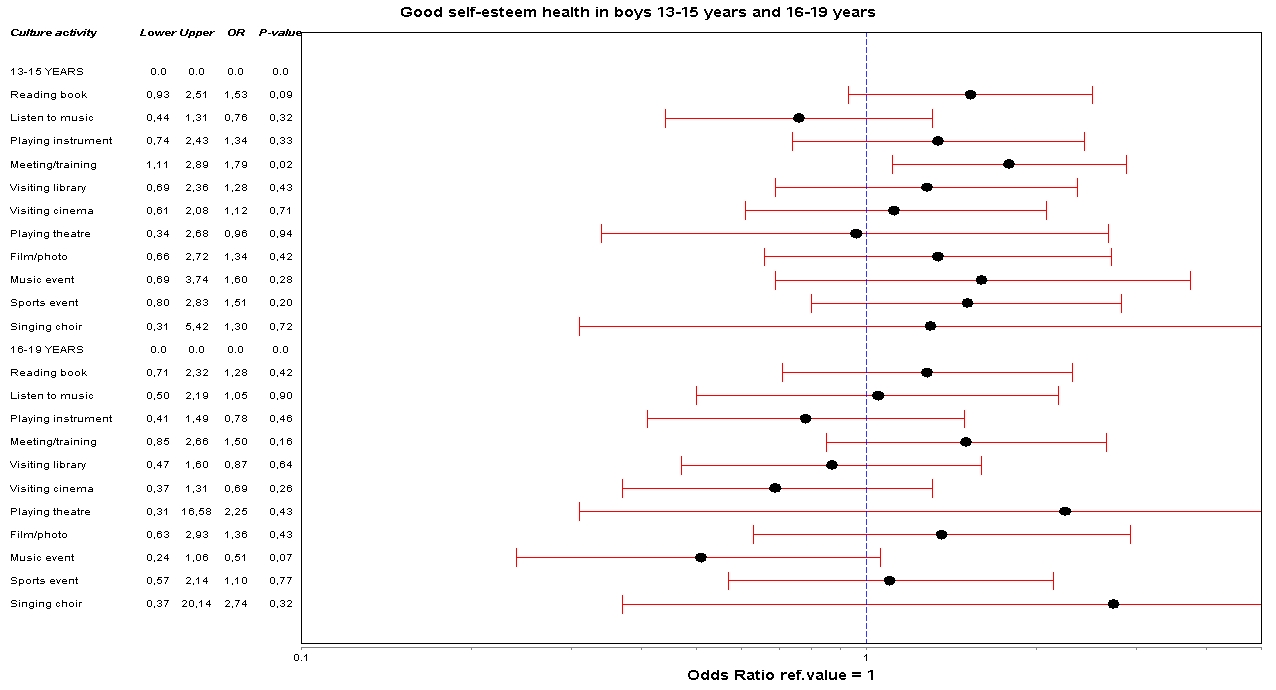


**Figure 4. Low anxiety/depression in girls and boys (13-15 years and 16-19 years old).**


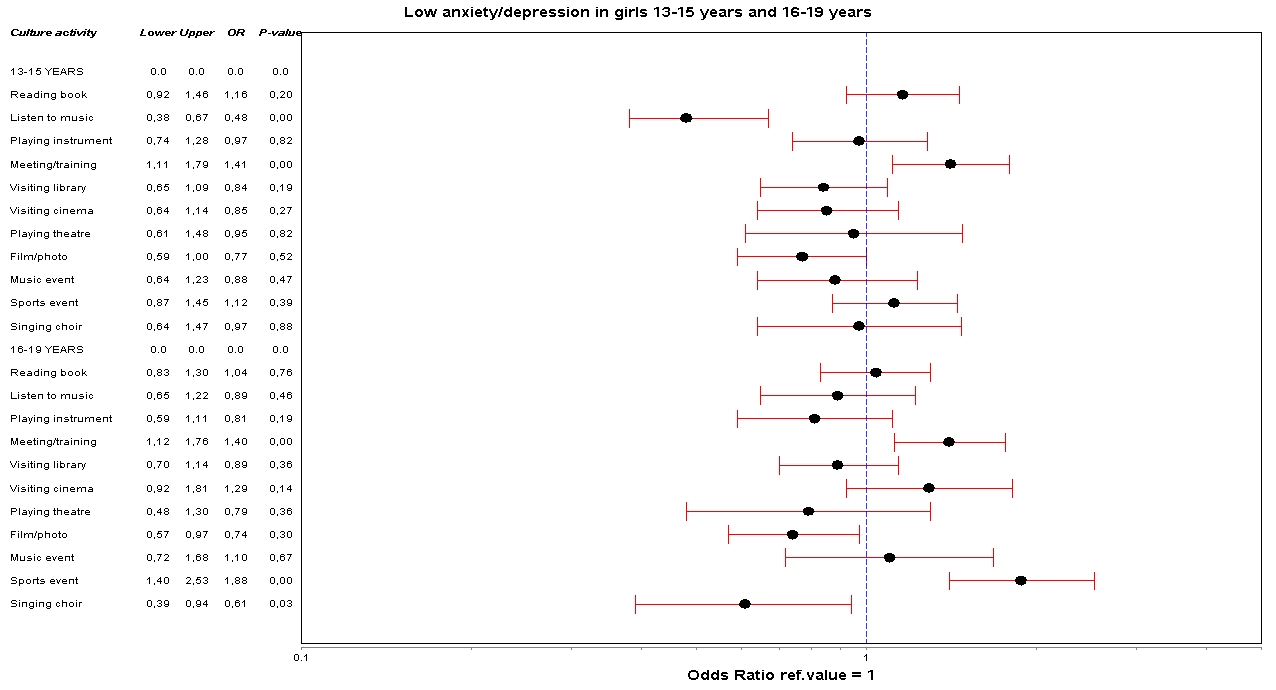


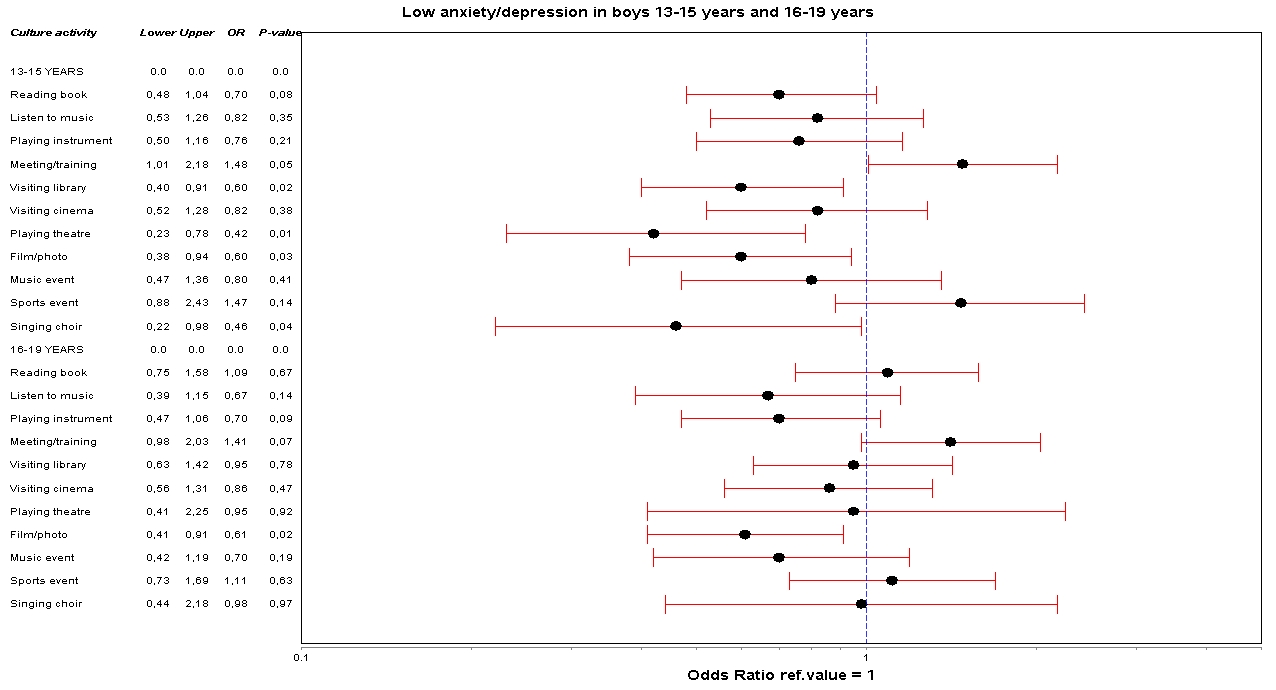

Supplement: Additional file 1: Figure S1. — Good Self-perceived health in girls and boys (13–15 years and 16–19 years old). Figure S2. Good Life-satisfaction health in girls and boys (13–15 years and 16–19 years old). Figure S3. Good Self-esteem health in girls and boys (13–15 years and 16–19 years old). Figure S4. Low anxiety/depression in girls and boys (13–15 years and 16–19 years old). [file 12889_2015_1873_MOESM1_ESM.docx]
